# Use HypE to Hide Association Rules by Adding Items

**DOI:** 10.1371/journal.pone.0127834

**Published:** 2015-06-12

**Authors:** Peng Cheng, Chun-Wei Lin, Jeng-Shyang Pan

**Affiliations:** 1 College of Computer and Information Science, Southwest University, Chongqing, P.R. China; 2 Shenzhen Graduate School, Harbin Institute of Technology, Shenzhen, Guangdong, P.R. China; Tokai University, JAPAN

## Abstract

During business collaboration, partners may benefit through sharing data. People may use data mining tools to discover useful relationships from shared data. However, some relationships are sensitive to the data owners and they hope to conceal them before sharing. In this paper, we address this problem in forms of association rule hiding. A hiding method based on evolutionary multi-objective optimization (EMO) is proposed, which performs the hiding task by selectively inserting items into the database to decrease the confidence of sensitive rules below specified thresholds. The side effects generated during the hiding process are taken as optimization goals to be minimized. HypE, a recently proposed EMO algorithm, is utilized to identify promising transactions for modification to minimize side effects. Results on real datasets demonstrate that the proposed method can effectively perform sanitization with fewer damages to the non-sensitive knowledge in most cases.

## 1 Introduction

It is common for data to be shared among different organizations during business collaboration. People may utilize data mining techniques to extract useful knowledge from shared data. However, despite its benefits to business or organization decision, data mining may pose the threat of disclosing sensitive knowledge to other parties. In such circumstances, it is necessary to ensure that not only private data such as the identifier or salary is preserved but also the sensitive knowledge behind a database is also not disclosed.

Hence, a problem arises how to balance the confidentiality of the sensitive knowledge with the legitimate mining need of data users. In this paper, we focus on privacy preserving in association rule mining. To illustrate the need of protecting sensitive rules before releasing data, we present the following scenario which is borrowed from the work of Menon et al. [1]. Suppose that two supermarkets which sell complementary products are interested to combine their data to identify potential relationships to improve profits. However, patterns contained in shared data may include business strategies and leakage of them to competitors may damage the interest of data owner. For instance, some strategies of allocating goods on shelf space may be profitable currently, and publishing data may disclose them to competitors. Therefore, the data owner hope to find some ways to conceal sensitive patterns.

To address this issue, a feasible solution is to transform the original database so that the sensitive rules in it cannot be mined out from a data mining view. However, transformation could lead to non-sensitive rules to be lost or new ghost rules to be generated. So the challenge is how to protect the sensitive rules while non-sensitive ones can still be mined out in the modified database to the largest extent. The transforming process from the original database into a modified one to protect sensitive knowledge is called as association rule hiding/data sanitization.

Since the database is changed, when applying the same mining algorithm with the same parameters on the modified database, obtained rules could be different. After sanitization, some rules may be lost or some new rules may be added in the modified database. In addition, the original data has been distorted. These side effects can be used to assess the performance of a sanitization approach. The ideal case is that all sensitive rules are completely hidden, at the same time no non-sensitive rules are lost and no ghost rules are newly generated. However, in most real cases, it is difficult to achieve such an ideal goal. A lot of effort has already headed towards the direction of minimizing side effects [2, 3]. However, an important fact has been neglected in the past literature. Actually, a tradeoff relation exists when minimizing side effects simultaneously, and improving one dimension often lead to degradation on other dimensions.

Based on the tradeoff characteristic existing within side effects, this study solves the rule hiding problem from the point view of multi-objective optimization. The technique of evolutionary multi-objective optimization (EMO) is used to solve it. The side effects are formulated as optimization goals to be minimized. The model we adopted to hide sensitive rules is to add items in some identified transactions so that sensitive rules’ confidences decrease and eventually escape the mining in the modified database at a predefined threshold. The Hype algorithm [4], a many-objective optimization evolutionary algorithm, is utilized to find a suitable subset of transactions for modification so that the damage to knowledge and data can be minimized. Through a set of experiments, we demonstrate the effectiveness of this approach. In addition, the proposed algorithm is robust with regard to datasets. In addition, it may hide multiple rules at one time. This work is an improvement of the study in [5]. Apart from the former three optimization goals, the data distortion was added into the optimization objective vector. Considering four optimization objectives, we adopted the HypE algorithm to find suitable candidates for sanitization.

The contribution of this work is summarized as follows. Firstly, it provides a new perspective on association rules hiding in consideration of the tradeoff relation within side effects. The EMO-based solution can deal with such a situation and produce multiple solutions in a single run. A user may freely choose a preferred one from them. Secondly, hiding methods by adding items have rarely been studied. This paper investigates the relation within side effects produced by adding items, and compares it with other hiding strategies. Experimental results show that the proposed approach may perform sanitization with fewer degrees of knowledge distortion in most cases.

The remainder of the paper is organized as follows. Section 2 introduces related work. Section 3 gives some basic concepts on association rule mining and multi-objective optimization. Section 4 formulates our research problem from the view of multi-objective optimization. The rule hiding algorithm based on EMO is presented in section 5. Section 6 shows experiment results and gives relevant performance discussions. Finally, we conclude this paper in Section 7.

## 2 Related Work

There has been a great deal of work on frequent itemset or association rule hiding. Atallah et al. [6] firstly proposed a greedy algorithm for itemset hiding and proved that the optimal solution to the underlying problem is NP-hard. Following this work, many algorithms were proposed. Dasseni and Verikios et al. [7, 8] proposed several heuristic hiding approaches to hide association rules by reducing their support or confidence levels. Among them, the algorithm 1.a hides rules by adding items to decrease sensitive rules’ confidence levels, and the other algorithms including 1.b, 2.a and 2.b hide sensitive rules or itemsets by deleting items.

Oliveira et al. [17] introduced an efficient sanitization algorithm based on the notion of disclosure threshold. This approach allows a database owner to specify a different threshold for each restrictive pattern. Amiri [12] proposed three heuristic algorithms to hide sensitive itemsets by removing transactions or items. The candidate transactions are identified in terms of the number of sensitive and non-sensitive itemsets they support. Verikios et al. [9] invented a heuristic hiding technique WSDA, which perform hiding by suppressing the confidence of a rule. Wu et al. [15] devised a template-based method aimed at avoiding all the side effects in rule the hiding process instead of hiding all sensitive rules.

Borrowing the concept of TF-IDF (Term Frequency-Inverse Document Frequency) in text mining, Hong et al. [11] devised a greedy-based hiding approach which assigns each transaction a SIF-IDF value to evaluate the correlation degree of the transaction with the sensitive itemset.

Sun [13] proposed a border-based approach, which focuses on preserving the border of non-sensitive frequent itemsets rather than considering all during the sanitization process. Menon et al. [1, 18] transformed the problem of frequent itemset hiding into a CSP and utilized integer programming techniques to solve it. Building upon the border theory [13], Moustakides et al. [16] proposed two algorithms which perform sanitization by maximizing the positive border itemsets above the frequency threshold. Divanis et al. [19] proposed an exact approach based on CSP and the border theory, which hides rules by extending the database.

All above algorithms are distortion-based, which perform the hiding task by removing or adding items in a dataset. In contrast, Saygin et al. [10] introduced an innovative blocking-based technique, which replaces some original values of a dataset with unknowns. The main difference of a blocking-based method with a distortion-based method is that it does not add any false information into the original dataset. This may be useful for some real-life applications. Pontikakis et al. [20] and Verikios et al. [9] developed the blocking-based technology to reduce the risk of attacks from an adversary.

Considering existing methodologies from different dimensions, their differences were summarized in Table 1. We propose a EMO-based solution to perform the rule hiding task by adding items, which may conceal sensitive knowledge with fewer side effect of knowledge distortion in most cases. The technique of evolutionary multi-objective optimization may well deal with the conflicting requirements of simultaneously minimizing various side effects.

**Table 1:** Difference of hiding solutions.

Method	Strategy of data modification		Knowledge form		Type of data modification	
	Distortion	Block	Rule	Itemset	Delete	Add
EMO-AddItem	✓		✓			✓
Algo1.a [7]	✓		✓			✓
Algo1.b, Algo2.a [7]	✓		✓		✓	
Algo2.b [7]	✓			✓	✓	
WSDA [9]	✓		✓		✓	
BA [9]		✓	✓		✓	✓
GIH [10]		✓		✓	✓	
CR [10]		✓	✓		✓	
CR2 [10]		✓	✓			✓
SIF-IDF [11]	✓			✓	✓	
Hybrid [12]	✓			✓	✓	
Border-based [13]	✓			✓	✓	
CSP-based [1, 14]	✓			✓	✓	
Template-based [15]	✓		✓		✓	✓
MaxMin [16]	✓			✓	✓	

## 3 Preliminaries

In this section, we introduce some basic notions about association rule mining and multi-objective optimization which are required to understand the rest of this paper.

### 3.1 Basic notions of association rule mining

Let *I* = {*I*_1_, *I*_2_, …, *I*_*m*_} be a set of items available. An itemset *X* is a subset of *I*. A transaction *t* is characterized by an ordered pair, denoted as *t* = <*ID*, *X*>, where *ID* is a unique transaction identifier number and *X* represents a list of items which the transaction contains. A transactional database *D* is a relation which consists of a set of transactions. For instance, in the market basket data, a transactional database is composed of business transactions. Each transaction consists of items purchased in a store. The absolute support of an itemset *X* is the number of transactions in *D* that contain *X*. Likewise, the relative support of *X* is the fraction (or percentage) of the transactions in a database which contain the itemset *X*, denoted as *Supp*(*X*). An itemset *X* is called frequent if *Supp*(*X*) is at least equal to a minimum relative support threshold (denoted as *MST*) specified by user. The goal of frequent itemset mining is to find all itemsets which are frequent. The notion of confidence is relevant to association rules. A rule has the form of *X* → *Y*. It means that the antecedent *X* infers to the consequent *Y*. Here both *X* and *Y* are itemsets. *X* ∩ *Y* = ∅. The confidence of a rule is computed as *Supp*(*X* ∪ *Y*)/*Supp*(*X*), and denoted as *Conf*(*X* → *Y*). It indicates a rule’s reliability. Like *MST*, the user also can define a minimum confidence threshold called *MCT*.

A rule *X* → *Y* is strong if it satisfies the following condition:

1. *Supp*(*X* ∪ *Y*) ≥ *MST* and
2. *Conf*(*X* → *Y*) ≥ *MCT*.

Association rule mining usually includes two phases: (1) Firstly, frequent itemsets are mined out with given *MST*. (2) Then, strong association rules are generated from the frequent itemsets obtained in phase 1 based on given *MCT*. In the following part of this paper, when the concept of association rules are used, we refer to strong rules if no special instructions.

**Fig 1:**
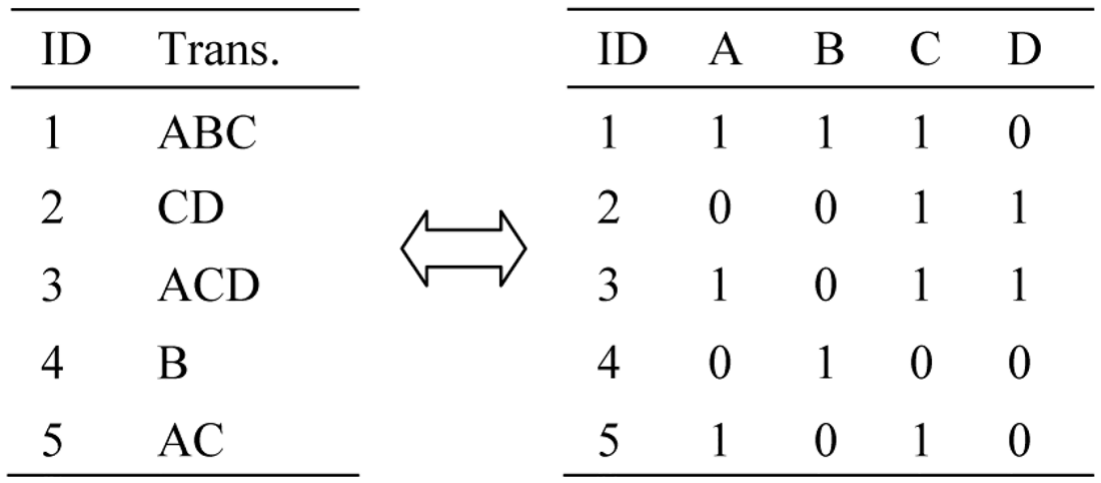
Conversion between transactional database and bit-vectors.

In order to ease the comprehensibility, we adopt the bit-vectors to express a transaction database, as indicated in Fig 1. Left side is a transaction database, which is represented as bit-vectors at right side. If *MST* = 50% and *MCT* = 80%, then the rule *A* → *C* is strong since *Supp*(*A*∪*C*) = 60% and *Conf*(*A* → *C*) = 100%.

### 3.2 Multi-objective optimization

The multi-criteria nature of association rule hiding problem brings inspiration for use of evolutionary multi-objective optimization (EMO) as a solution. A multi-objective optimization problem (MOP) has two or more usually conflicting objectives that need to be optimized simultaneously. Because of this nature, a MOP often has several optimal solutions rather than a single one. Here we define formally the concept of MOP, Pareto dominance, Pareto optimality, Pareto optimal set and Pareto front.

**Definition 3.1.** *MOP*

Without loss of generality, assuming that minimization is the goal for all objectives. A MOP has the following form:

Minimize: $\bar{f}(\bar{x})=[f_{1}(\bar{x}),f_{2}(\bar{x}),...,f_{n}(\bar{x})]$

Subject to: $\begin{array}{cc} & g_{i}(\bar{x})\leq 0,i=1,2,\ldots ,m\\ & h_{j}(\bar{x})=0,j=1,2,\ldots ,p \end{array}$

Where $\bar{x}={\left[x_{1},x_{2},\ldots ,x_{n}\right]}^{T}$ is a vector of decision variables, $\bar{f}$ is a vector of *k* objective functions. *g*_*i*_ and *h*_*j*_ are the inequality and equality constraint functions of the problem. The set of all solutions satisfying the constraints defines the feasible space Ω.

**Definition 3.2.** *Pareto Domination*

A solution $\bar{x}={\left[x_{1},x_{2},\ldots ,x_{n}\right]}^{T}$ is said to dominate another solution $\bar{y}={\left[y_{1},y_{2},\ldots ,y_{n}\right]}^{T}$, if and only if ∀*i* ∈ {1, 2, …, *n*} : *x*_*i*_ ≤ *y*_*i*_ ∧ ∃*i* ∈ {1, 2, …, *n*} : *x*_*i*_ < *y*_*i*_, denoted as $\bar{x}≺\bar{y}$.

**Definition 3.3.** *Pareto Optimality*

A solution $\bar{x^{*}}$ is said to be Pareto optimal if there does not exist another $\bar{x}$ such that $\bar{x}≺\bar{x^{*}}$.

**Definition 3.4.** *Pareto Optimal Set*

For a given MOP, the Pareto optimal set is defined as $PS^{*}=\{\bar{x}\in \Omega |\neg \exists \bar{x^{\prime }}\in \Omega ,\bar{x^{\prime }}≺\bar{x}\}$.

**Definition 3.5.** *Pareto Front*

For a given MOP, assuming P* is its Pareto optimal set, the corresponding Pareto front is defined as $PF^{*}=\{\bar{f}(\bar{x})|\bar{x}\in PS^{*}\}$.

The main goal of evolutionary multi-objective optimization is to find the Pareto front for a give MOP. In practice, it only needs to find an approximation set as close as possible to the true Pareto front and distributed uniformly and widely.

## 4 Problem Formulation

The sensitive rules hiding problem can be formulated as follows. Let *D* be a transactional database and *R* be the set of strong rules that can be mined from *D* with given *MST* and *MCT*. Let *R*_*S*_ denote a set of sensitive rules that need to be hidden, and *R*_*S*_ ⊂ *R*. *R*_*N*_ is the set of non-sensitive rules. *R*_*N*_ ∪ *R*_*S*_ = *R*. The hiding process is to transform *D* into a sanitized database *D*′ such that only the rules which belong to *R*_*N*_ can be mined from *D*′. ∣*D*∣ = ∣*D*′∣. Let *R*′ denote the strong rules mined from sanitized database *D*′ with the same *MST* and *MCT*.

Let Δ be the transactions which are identified to be modified in database *D* in order to conceal sensitive rules. Then Δ ⊆ *D* and Δ = *D* − *D*′. ∣Δ∣ denotes the number of modified transactions. Let Δ′ be the transactions which have made changes in *D*′. Then Δ′ ⊆ *D*′ and Δ′ = *D*′ − *D*. ∣Δ∣ = ∣Δ′∣. A hiding approach finds the set Δ and transforms Δ into Δ′.

Side effects may be generated after transforming *D* into *D*′. The sensitive rules which are not hidden in the modified database *D*′ are called as S-N-H(Sensitive rules Not Hidden). S-N-H = {*r* ∈ *R*_*S*_∣*r* ∈ *R*′}. The non-sensitive rules, which are falsely hidden and lost in the modified database *D*′, are represented as N-S-L (Non-Sensitive rules Lost). N-S-L = {*r* ∈ *R*_*N*_∣*r* ∉ *R*′}. The rules falsely generated in sanitized database *D*′ is marked as S-F-G (Spurious rules Falsely Generated). S-F-G= {*r* ∈ *R*′∣*r* ∉ *R*}.

The goal of a hiding method is to hide the sensitive rules with side effects as fewer as possible. In other words, the ideal result is that all three sets, i.e. S-N-H, N-S-L and S-F-G, are empty. In practice, side effects always occur along with the sanitization process. To select different transactions subset for modification will result in accordingly different side effects.

Another impact produced in the sanitization process is data distortion. Since the data is intended to be shared, it is important to conceal sensitive rules with minimal damage to the database itself. The degree of data distortion can be measured as the proportion of transactions that are sanitized. On the base of the above discussion, we can formulate the sensitive rules hiding task as a multi-objective optimization problem as showed in Eq 1. All objective functions are normalized into the interval [0, 1].

Minimize $\bar{f}=[f_{1},f_{2},f_{3},f_{4}]$

$$\begin{array}{ccc} & & f_{1}=\left|S-N-H|/\right|R_{S}|\\ & & f_{2}=\left|N-S-L|/(|R|\;\right|R_{S}\left|\right)\\ & & f_{3}=\left|S-F-G|/|R\right|\\ & & f_{4}=\left|X\right|/\left|D\right| \end{array}$$ (1)

Theoretically, the size of search space is {1, 2, …, *n*}^*k*^. Where *n* is the database size and *k* is the number of transactions to be sanitized. The search space is often very huge. However, the algorithm only needs to find candidates within the scope of supporting transactions of sensitive rules.

## 5 Proposed Solution

The proposed approach hides sensitive rules by inserting new items into some identified transactions so as to decrease their confidence values below MCT. Two key issues need to be solved for this modification.

1. Find suitable transactions for modification in the database.
2. Determine which items to be inserted in an identified transaction.

Most existing association rule hiding approaches adopt deterministic means to settle the above both problems. In our method, EMO is used to solve the first problem, i.e., to identify suitable candidate transactions for modification, while an exact method is adopted to solve the second problem when the candidate transactions have been determined.

### 5.1 Basic hiding strategies

The model we adopted to hide it is to add/insert new items into a dataset to decrease the confidence of the rule below *MCT* [5]. In order to reduce the confidence of a sensitive rule, the transactions, which partially support the rule’s antecedent but not fully support consequent, can be selected for modification by adding new items to make it fully support the antecedent. By this way, the support of antecedent is increased while the support of the generating itemset of the rule remains unchanged. The rule’s confidence will descend since the confidence is calculated as the support of the generating itemset of the rule divided by the support of the antecedent part.

To hide sensitive rules based on the above strategies, the operation needs to satisfy the following properties. Similar proofs for this property appeared in [7, 15].

**Theorem 5.1.** *Assume that X → Y is a sensitive rule to be concealed. Let Σ_X_ be the set of all transactions which partially (or not fully) support the rule’s antecedent X and not fully support the consequent Y. To hide X → Y by inserting items into the transaction in Σ_X_ to make it fully support the antecedent X, the minimal number of transactions that should be modified is:*

$$\begin{array}{c} NUM_{add}\;=\;\left\lceil Supp(X\;\cup \;Y)*\;|D|\;/MCT\;-\;Supp(X)\;*\;|D|\right\rceil \;+\;1 \end{array}$$ (2)

**Proof.** Inserting items into a transaction in Σ_*X*_ to make it fully support the rule’s antecedent *X* will raise the support of the antecedent by 1. Assume that *θ* is the minimal number of transactions in Σ_*X*_ which need to be modified to fully support *X* in order to reduce the confidence of the rule below *MCT*. Then we may get:

$$\begin{array}{ccc} & & (Supp(X\;\cup \;Y)\;*\;|D|)/(Supp(X)\;+\;\theta )<MCT\\ \to \; & & Supp(X\;\cup \;Y)\;*\;|D|/MCT-Supp(X)\;*\;|D|<\theta \end{array}$$

Because *θ* is an integer and *θ* is the minimum number which is greater than *Supp*(*X* ∪ *Y*) * ∣*D*∣/*MCT* − *Supp*(*X*) * ∣*D*∣, we may get:

$$\begin{array}{ccc} & & \theta >Supp(X\;\cup \;Y)\;*\;|D|/MCT-Supp(\;X\;)\;*\;|D|\\ \to \; & & \theta \;=\;\lceil Supp(X\;\cup \;Y)\;*\;|D|/MCT-\;Supp(X)\;*\;|D|\rceil \;+\;1 \end{array}$$

Note that the minimum number of sanitized transactions specified by Eq 2 cannot ensure that all sensitive rules to be hidden in some situations. Two special cases must be taken into account.

1. It is possible that ∣Σ_*X*_∣ is less than *NUM*_*add*_. If this happens, then there are no sufficient candidate transactions for modification to reduce the confidence of the sensitive rule. ∣Σ_*X*_∣ denotes the number of available transactions which partially support the antecedent part of the sensitive rule *X* → *Y*.
2. It is possible that there are common items between different sensitive rules. It is a situation often encountered if a greater number of rules are specified as sensitive. A large number of sensitive rules increase the possibility of overlapping items existing within them. Overlapping may lead to the occurrence of the following circumstance. To hide a subsequent sensitive rule, the transaction selected for modification may support only the antecedent of another already hidden sensitive rule with overlapping items. The modification may cause the increase of the confidence of the overlapping rule and make it to be exposed again.

### 5.2 Algorithm EMO-AddItem

After determining the basic hiding strategy, the challenging issue is how EMO can be utilized to find suitable transactions to modify (by adding items) so as to minimize side effects simultaneously. In this section we firstly introduce the general procedure of a hiding process, which was described previously in [5]. The general procedure is named as EMO-AddItem as showed in Algorithm 1. The principle of adapting EMO to find candidate transactions for modification will be discussed in detail in the next section.

**Algorithm 1** EMO-AddItem

**Input:** the source database *D*, *MST*, *MCT*, and sensitive rules sets *R*_*S*_.

**Output:** the sanitized database *D*′ in which all sensitive rules cannot be mined out.

 Find frequent item sets and association rules in *D* using the improved Apriori algorithm.

**for** *i* = 1 to ∣*R*_*S*_∣ **do**

  Get the *i* ^*th*^ sensitive rule *r*_*i*_ : *X* → *Y*. *r*_*i*_ ∈ *R*_*S*_

  Σ_*i*_ ≔ {*t* ∈ *D*∣*X* ⊈ *t* ∧ *Y* ⊈ *t*}.

*NUM*_*i*_ = ⌈*Supp*(*X* ∪ *Y*)* ∣*D*∣/*MCT* − *Supp*(*X*) * ∣*D*∣⌉ + 1.

  // *Calculate the minimal number of transactions to be modified according to* Eq 2.

  The length of the *i* ^*th*^ chromosome segment ≔ *NUM*_*i*_.

**end for**

*T* ≔ EMO_find().

 // *utilize EMO to find transaction set *T* to modify to hide sensitive rules while*.

 // *minimizing side effects. T is divided into s parts: T_1_, T_2_, …, T_s_, s = ∣R_S_∣*.

 // *T_i_ contains selected transactions which partially support the antecedent of *r*_*i*_*.

 // *∣*T*_*i*_∣ = *NUM*_*i*_ and *T* = *T*_1_ ∪ *T*_2_ ∪ … ∪ *T*_*s*_*.

**for** *i* = 1 to ∣*R*_*S*_∣ **do**

**for** each transaction *t* ∈ *T*_*i*_ **do**

   Adding items into *t* to make it fully support the antecedent of rule *r*_*i*_.

**end for**

**end for**

The algorithm EMO-AddItem hides sensitive rules by increasing the support values of their antecedents until the rules’ confidence values get below *MCT*. Firstly, it uses an improved version of the Apriori algorithm [21–23] to mine out frequent itemsets and association rules. Some recent association rule mining algorithms also may be referenced, such as works in [24, 25]. For each sensitive rule *r*_*i*_, the transactions, which partially support the antecedent but not fully support the consequent part, are filtered out and their transactions IDs are stored in Σ_*i*_. According to Eq 2, the minimum number of transactions which have to be sanitized to hide the *i* ^*th*^ sensitive rule, i.e. *r*_*i*_, is calculated, which can be used to decide the length of *i* ^*th*^ segment in the chromosome encoding scheme (introduced in the later section).

Then, EMO is utilized to find suitable candidate transactions from Σ_*i*_ for modification. The EMO algorithm maintains a set of solutions in each iteration. It assesses the solution quality based on the generated side effects if the inserting operations according to the solution take place on the selected transactions. When the optimal solution is found, it is used to make a real modification on the database *D*. The optimal solution means that it can hide sensitive rules with the minimum side effects. The set of chosen transactions *T* is divided into ∣*R*_*S*_∣ parts, i.e., *T*_1_, *T*_2_, …, *T*_*s*_, *s* = ∣*R*_*S*_∣. And each part is related to a distinct sensitive rule, i.e., part *T*_*i*_ contains selected transactions which partially support the antecedent of the rule *r*_*i*_. If a transaction in *T*_*i*_, namely *t*, is modified by inserting new items so that it can fully support the antecedent part of *r*_*i*_ but not support the whole rule, the confidence of the rule *r*_*i*_ will decrease. When all the transactions in *T*_*i*_ have been modified in such a manner, the sensitive rule *r*_*i*_ can be hidden because its confidence gets below *MCT*. However, the exception may occur. For instance, if there are common items between different sensitive rules, some sensitive rules could not be concealed.

In order to improve the efficiency, the original database was transformed into a “thin” database. A “thin” database means that only frequent items are stored in memory for each transaction and the non-frequent items are discarded. Thus, each transaction in a “thin” database became shorter. The “thin” database is kept in memory and the use of it can bring the benefit that both the memory space and scanning time can be reduced greatly since the size of each transaction has become shorter.

### 5.3 Use EMO to find candidate transactions for modification

Evolutionary multi-objective optimization (EMO) refers to use the evolutionary algorithm to solve multi-objective optimization problems. The population-based characteristic of evolution computation make it very suitable to solve multi-objective optimization problem. In addition, the heuristic nature of evolution algorithms makes them robust to the specific features of a MOP. As fast growing in this field, many evolutionary multi-objective optimization algorithms have been proposed [26–28]. For instance, NSGA-II [29], SPEA2 [30] and other Pareto-dominance based EMO algorithms have been successfully employed in various bi-objective optimization problems.

However, the Pareto-dominance based EMO methods have been found to scale poorly when the number of optimization goals increases. Researchers tried to find alternatives solve the problem. Recently, the hypervolume-based methods [4, 31] have become popular because the hypervolume indicator is the only known quality measure which is strictly monotonic with Pareto dominance and is shown to have a good adaptability to the many-objective optimization problem. The main barrier to utilize the hypervolume is its high computational requirement. To address this issue, the HypE [4] has been proposed which uses Monte Carlo simulation to approximate the exact hypervolume values.

In particular, a platform and programming language independent framework—PISA [32] has been developed to ease comparing and applying various EMO algorithms. In our study, we implement the rule hiding solution based on the PISA framework. For the rule hiding problem with four optimization goals (as indicated in Eq 1), we adopted HypE as the selector part of EMO in the PISA framework. The problem-related encoding scheme and variation operators were devised for the variation part to efficiently conduct the search in the huge decision space. We will discuss them in detail in the following section.

#### Encoding scheme for EMO

In order to reduce the search space and improve the efficiency, we devised a problem-realted encoding scheme. The chromosome consists of IDs of selected transactions to be modified. Each gene on a chromosome represents an ID of one selected transaction. The chromosome is divided into several segments. Each segment is related to a distinct sensitive rule and it selects genes only from the IDs of transactions which partially (not fully) support the antecedent of corresponding sensitive rule. Assuming there are *n* sensitive rules to be hidden, and then the chromosome includes *n* segments. The length of the *i* ^*th*^ segment in the chromosome is determined by the Eq 2.

**Fig 2:**
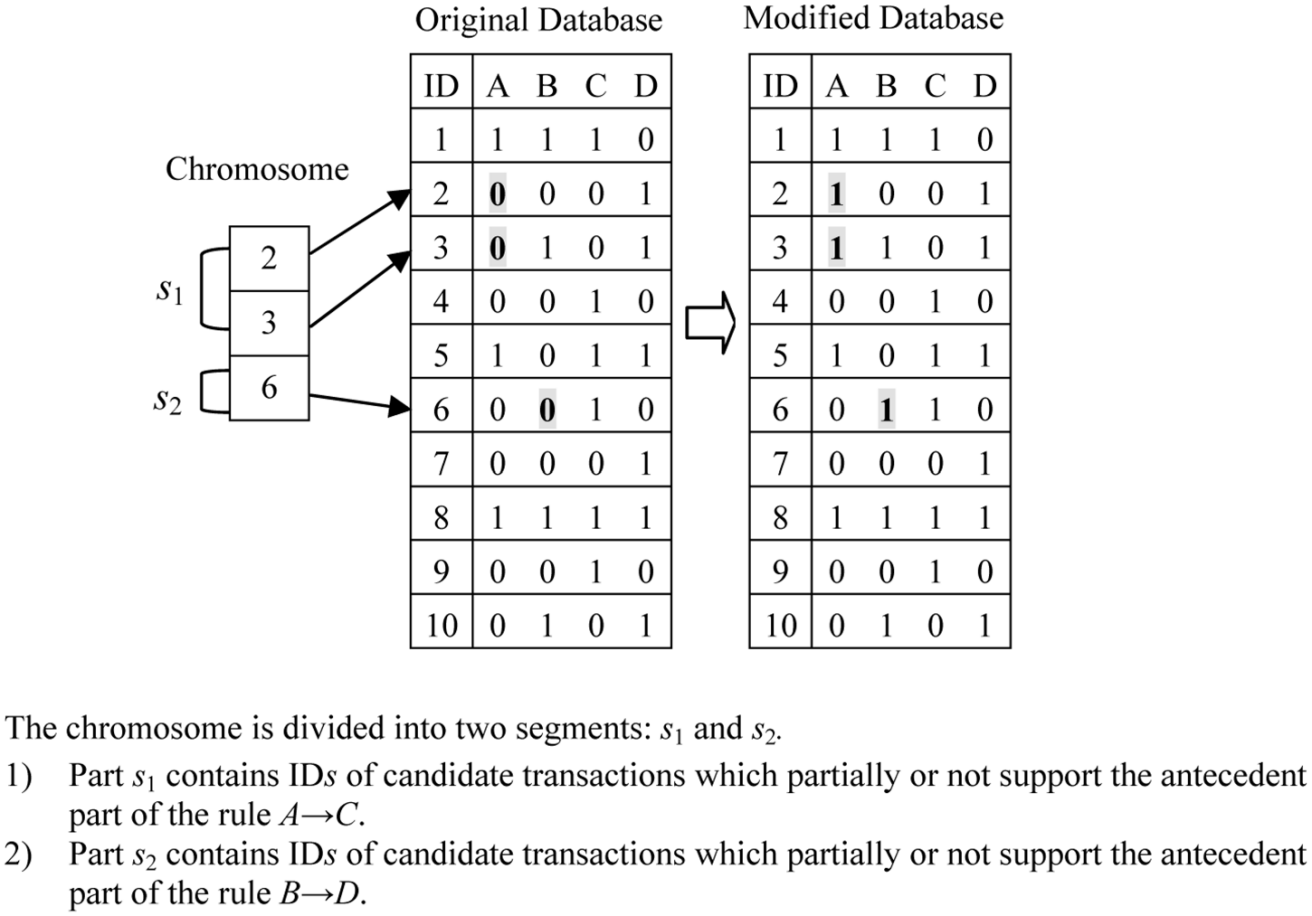
The mechanism of chromosome encoding.

Fig 2 introduces an example which illustrates the principle of encoding which can hide sensitive rules by adding items. Assume *MST* = 20% and *MCT* = 70%. There are 10 transactions in the original database. We are interested to hide the rule *A* → *C* and *B* → *D*. For the rule *A* → *C*, the collection of IDs for transactions, which not fully support the item *A* and not fully support the item *C*, is {2, 3, 7, 10}. Similarly, for the rule *B* → *D*, the relevant collection of IDs is {4, 6, 9}.

As Fig 2 indicates, the IDs contained in the chromosome are {2, 3, 6}. The chromosome is divided into two parts. {2, 3} belongs to the part *s*_1_ and {6} belongs to the part *s*_2_. *s*_1_ and *s*_2_ are related to the sensitive rule *A* → *C* and *B* → *D* respectively.

According to the chromosome, the transactions with IDs in {2, 3, 6} need to be sanitized. New items are inserted into these transactions by transforming some selected items from value 0 to value 1, as indicated in Fig 2. Before modification, *Conf*(*A* → *C*) = 100%. If the 2^*nd*^ and 3^*rd*^ transactions are selected to turn to 1s the elements corresponding to the attribute *A*, then *Conf*(*A* → *C*) = 60% in the modified database. Hence the rule *A* → *C* becomes hidden (not strong) after the data modification.

For the rule *B* → *D*, before modification, *Conf*(*B* → *D*) = 75%. If the 6^*th*^ transaction is selected to turn into 1 the element corresponding to the attribute *B*, then *Conf*(*B* → *D*) = 60% and the rule *B* → *D* also will become hidden.

A sensitive rule may become hidden if a sufficient amount of supporting transactions is modified to reduce its confidence below *MCT*. However, for a sensitive rule *X* → *Y*, although modifying any transaction in Σ_*X*_ (Σ_*X*_ denotes the set of transactions which partially support the antecedent and not fully support the consequent) can decrease its confidence, it is still necessary to make a choice within the candidate set Σ_*X*_. This is because modifying different subset may cause different side effects. The main task of EMO is to find an appropriate subset on which modification can hide sensitive rules but causes the minimal side effects at the same time.

#### EMO procedure

Algorithm 2 indicates how to find out suitable transactions subset for modification using the HypE algorithm.

Firstly, the initial population is generated randomly. Each individual in the initial population is composed of ∣*R*_*S*_∣ encoding segments. The genes in the *i* ^*th*^ segment come from IDs of the randomly chosen supporting transactions in Σ_*i*_ for the sensitive rule *r*_*i*_. These individuals’ objective function values are calculated according to how much side effects they may bring. The module of calculating objective functions is important because it may greatly affect the performance.

In the evolutionary process, *λ* new individuals are generated by variation operators in each iteration. Every newly generated offspring is assigned an objective vector based on calculation of the objective function. Then, the offsprings are merged with *μ* parent individuals. HypE is used to assign a fitness value for each individual and select out new *μ* optimal ones from (*μ* + *λ*) solutions. The *μ* optimal individuals will be kept into the next generation. At last, the mating selection is performed to choose parent individuals for the next generation. This procedure is iterated until the maximal generation is reached.

**Algorithm 2** EMO_Find

**Input:** the source database *D*, *MST*, *MCT*, the sensitive rules set *R*_*S*_ and supporting transactions set Σ = Σ_1_ ∪ Σ_2_ ∪ … ∪ Σ_∣*R*_*S*_∣_.

**Output:** the identified transaction set *T* to be modified.

*P*_0_ ≔ Generate the initial population, *t* ≔ 0.

**for** each individual *x* ∈ *P*_0_ **do**

  Call *Eval*(*x*) to calculate the objective function values.

**end for**

**repeat**

*Q*_*t*_ ≔ *Generate*(*P*_*t*_).

**for** each individual *x* ∈ *Q*_*t*_ **do**

   Call Eval(x) to calculate objective values.

**end for**

*P*_*t*_ ≔ (*P*_*t*_ ∪ *Q*_*t*_).

  Fitness assignment and sorting using HypE.

  Selection: reduce *P*_*t*_ from (*μ* + *λ*) to *μ*.

  Mating selection (*P*_*t*_).

*t* ≔ *t*+1.

**until** the max generation is reached

 Choose the preferred solution from the Pareto optimal set.

#### Variation operators

As discussed previously, genes of the *i* ^*th*^ segment of a chromosome should be composed of IDs from Σ_*i*_. Σ_*i*_ is the set of transactions which partially (or not fully) support the *i* ^*th*^ rule’s antecedent and not fully support the consequent. The variation operators should satisfy this limitation and ensure the genes of offspring to be valid. Common crossover operators, like single-point, multi-point or uniform crossover, cannot guarantee to generate valid offsprings, because they cannot ensure that the *i* ^*th*^ segment of an offspring chromosome is still composed of IDs from Σ_*i*_, or possesses a valid (sufficient) number of IDs of transactions from Σ_*i*_ to hide *i* ^*th*^ sensitive rule. In order to solve this problem, we devised a shuffle crossover and a shuffle mutation operator.

**Fig 3:**
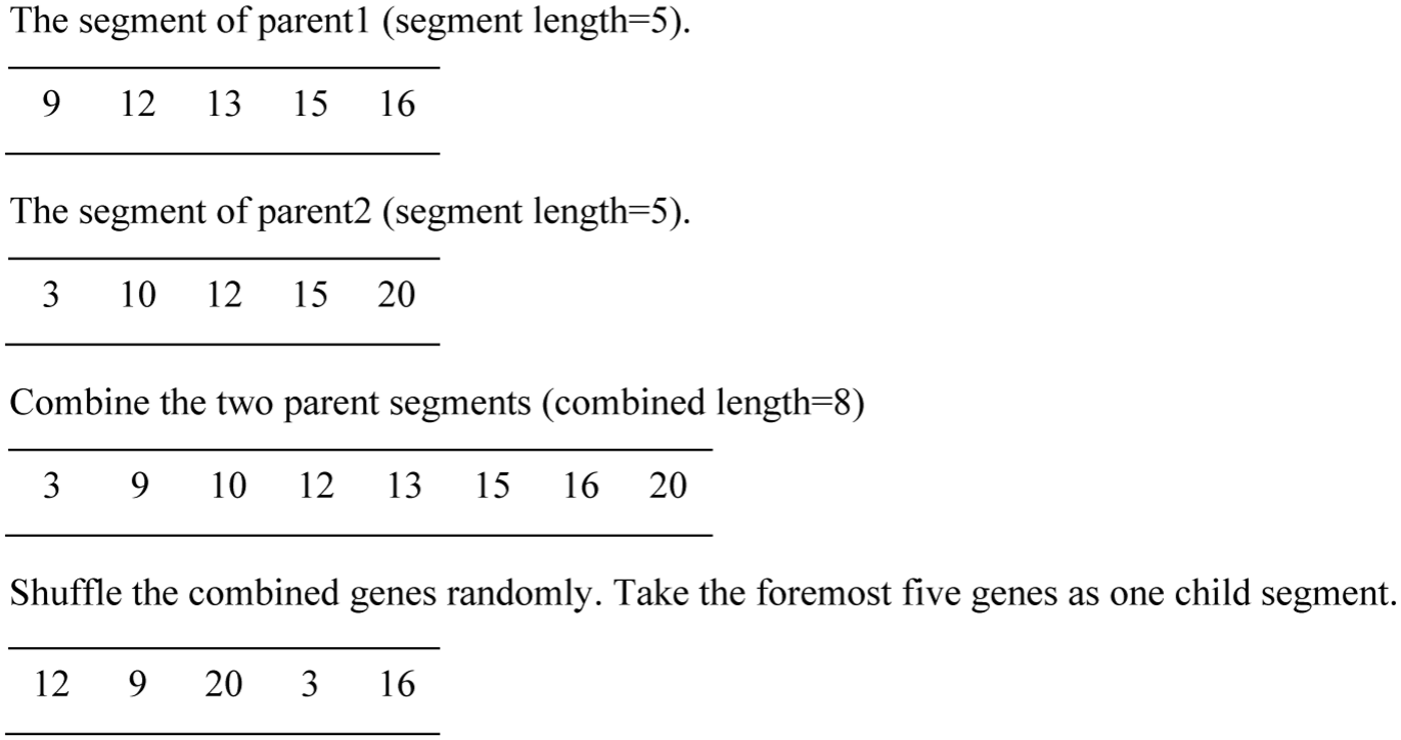
The shuffle crossover.

Since different solutions have the same number of segments in the chromosome, each segment of one parent solution may only performs crossover with the corresponding section of another parent at the same position. Supposing there are two parent segments at the same location of two individuals, the crossover operation between these two sections is illustrated as Fig 3.

The mutation operator also needs to satisfy the limitation that the *i* ^*th*^ chromosome section of offspring still is composed of IDs from Σ_*i*_ and the number of genes in the *i* ^*th*^ segment is valid (sufficient) to hide the *i* ^*th*^ sensitive rule. For the *i* ^*th*^ chromosome section of the offspring, original IDs are discarded and new IDs are randomly selected from Σ_*i*_.

#### Strategy of objective function calculation

The fitness of each individual is evaluated based on its objective functions vector. Each dimension of objective function vector corresponds to a specific type of side effect. The way of calculating objective functions may affect the efficiency greatly. There are two ways to calculate side effects for a solution.

1. Modify the corresponding transactions according to the solution’s genotype, and mine out new rules from the modified database. Then compare the original rule sets and new rules sets in order to determine the side effects. It seems to be infeasible since EMO needs to iteratively evaluate multiple solutions in each generation. This way requires to scan the database in each iteration and perform the mining task whenever evaluating a solution. It will be very time-consuming and is impossible to be applied in the real scenario.
2. Make a copy for the itemsets mined from the original database and make a copy for the “thin” database. According to a solution’s genotype, modify the relevant transactions in the database copy and then update the support of affected itemsets. After the modification is complete, update the supports of itemsets in the trie tree [23] (The algorithm adopts the trie tree to store frequent itemsets and their support values). The quantities of side effects can be obtained by comparing the original support and confidence with the new support and confidence for each rule. This option does not require repeated database scans and only updates the supports of itemsets. So it is efficient and feasible.

## 6 Performance Evaluations

The proposed approach was implemented in C++ based on the PISA platform [32] and ran on an Intel Xeon CPU X5650 with four 2.67GHz processors and with 4 GB of main memory. We carried out extensive experiments on real datasets. The experiment results were measured according to side effects on knowledge and data as follows (Note: the relevant notations are defined in Section 4). The lower the values on these metrics, the better a sanitization approach.

1. Hiding failure: it refers to the proportion of sensitive rules which fail to be hidden, calculated as ∣S-N-H∣ / ∣*R*_*S*_∣.
2. Knowledge distortion: it is the cumulative sum of the proportion of missing non-sensitive rules and the proportion of ghost rules, calculated as ∣N-S-L∣ / (∣*R*∣-∣*R*_*S*_∣) + ∣S-F-G∣ / ∣*R*∣.
3. Data loss or data distortion: it denotes the proportion of sanitized transactions, calculated as ∣Δ∣/∣*D*∣.

Generally, the evolutionary parameters are set empirically in the community. The parameters for EMO were specified as follows. The population size was 40 to ensure the diversity in the population. Considering the convergence speed, the maximal generation for evolution was 100. Experiments show that this can lead to the convergence in most test cases. The crossover and mutation probability were set as 0.95 and 0.1 respectively to balance the exploring and exploiting ability. For each test case, we ran the proposed algorithm for twenty times to get the average result.

The evolutionary multi-objective optimization algorithm can produce multiple Pareto optimal solutions on a single run. In the absence of additional preference information, none of the Pareto-optimal solutions can be said to be inferior to any other ones [33]. Choosing a preferred one involves many non-technical high-level information, and it is often experience-driven [27, 34].

For the problem of association rule hiding, solutions which do not reveal any sensitive rules but miss some non-sensitive ones or generate some ghost ones, are more preferred than solutions which reveal a few sensitive rules but produce no or fewer missing non-sensitive ones and ghost rules. Such preference is observed as sensitive rules are usually critical information which cannot be tolerated to be revealed even a few. So we give the first priority to the hiding failure when choosing a preferred solution. If the performance on hiding failure makes no difference, we still need to balance between side effects on knowledge distortion with the side effect on data distortion. As Amiri [12] emphasized, the primary goal of data sanitization is to conceal all sensitive rules (or itemsets) while the number of non-sensitive ones can be maintained to the maximal degree. The information on data accuracy (or data distortion) is only used for illustration. This point conforms to our views. Thus, if the hiding failure cannot differentiate solutions, the knowledge distortion will be attached more importance, over the degree of data distortion.

### 6.1 Datasets

We tested the proposed algorithm on four representative real databases, as indicated in Table 2. The Mushroom dataset was prepared by Roberto Bayardo [35]. Bms -1 and Bms- 2 were from Blue Martini Software incorporation. They were used for the KDD Cup of 2000 [29], and contain click stream data from the website of a legwear and legcare retailer. The Retail dataset was taken from an anonymous Belgian retail store and reported in [36]. All of them are publicly available through the FIMI repository located at http://fimi.cs.helsinki.fi/. These datasets exhibit varying characteristics with respect to the number of transactions and items that they contain, as well as with respect to the average transaction length. They were summarized in Table 2.

**Table 2:** Characteristics of real datsets and parameter settings.

Dataset	# Tran.	# Items	Avg. Tran. Len.	MST	MCT	# Freq. Itemsets	# Strong Rules
Mushroom	8124	119	23	5%	50%	1329	1065
Bms-1	59602	497	2.5	0.1%	20%	3065	3207
Bms-2	77512	3340	5.0	0.2%	20%	1196	1598
Retail	88162	16469	10.3	0.1%	50%	5054	3276

The value of *MST* depends on the density of a sanitized dataset. As Amiri [12] indicated, lower minimal support levels could be used with sparser datasets. Here, the density of a dataset is measured as the average transaction length divided by the number of available items. A denser dataset means that the average support of itemsets contained in it is higher and the *MST* level can be set relatively greater to control the explosion of frequent itemsets. In contrast, the average support of itemsets contained in a sparser dataset is lower and the *MST* level can be tuned smaller to ensure that a great number of itemsets can be generated. The level of *MCT* may decide the number of strong rules generated from frequent itemsets. A lower *MCT* value may produce more rules from frequent itemsets.

In our experiments, *MST* was set as follows. For the Mushroom dataset, it is much denser compared with other datasets, and we adopted 5% as its *MST* value. The Retail, Bms-1 and Bms-2 datasets are relatively sparser, and the *MST* levels of them were set as 0.1%, 0.1% and 0.2% respectively. When the *MST* levels were determined, *MCT* levels were appropriately selected for each dataset to ensure that sufficient strong association rules can be produced, as indicated in Table 2. Unless stated otherwise, these settings on *MST* and *MCT* for each dataset will be used in the following experiments.

### 6.2 Results and analysis

We compared the proposed method with the algorithm 1.a in [7], WSDA in [9] and SIF-IDF in [11]. The algorithm 1.a is such a method which performs the hiding task by adding items into a database. This modification strategy is similar to our method. Both algorithms aim at increasing the support of the antecedent part of a sensitive rule by adding new items, and accordingly decrease the confidence of the sensitive rule below *MCT*. WSDA hides a rule by removing items in supporting transactions until its confidence drops below *MCT*. It assigns a weight to each rule according to how far its confidence from *MCT*. The priority of a transaction is computed according to the sum of weights of rules contained in it. The weaker transactions are modified first. SIF-IDF hides a rule by removing items to suppress its support below *MST*. It selects sanitized transactions based on their relations to sensitive items.

Two series of experiments were conducted to investigate the performance. In the first series of tests, we examined the performance with different numbers of sensitive rules on four real datasets. 10 and 20 strong rules were selected randomly as sensitive ones for each dataset. Four algorithms were performed on eight test cases. In the second series of tests, only Bms-1 was used to explore the effects of different *MCT* levels. We selected 10 rules with high confidence values as sensitive ones to ensure that they were suitable to various *MCT* levels. The experiment result data is included in the Supporting Information section.

#### Results with increasing numbers of sensitive rules

The results are shown in Table 3. As expected, to hide more sensitive rules the sanitization process produces more side effects since it has to sanitize more transactions to conceal them. Methods based on removing items, i.e. WSDA and SIF-IDF can hide sensitive rules completely in most cases, in contrast to methods based on adding items. However, this is often achieved by greater knowledge distortion degrees.

**Table 3:** Results with the increasing size of sensitive rules.

			Side effects		
Dataset	∣*R*_*S*_∣	Method	Hiding failure(%)	Knowledge distortion(%)	Data distortion(%)
Mushroom	10	EMO-AddItem	20.000	2.449	49.489
		Algo1.a	20.000	5.087	36.234
		WSDA	0.000	2.935	36.148
		SIF-IDF	0.000	8.431	26.105
	20	EMO-AddItem	15.000	7.116	75.840
		Algo1.a	15.000	13.244	47.532
		WSDA	0.000	8.793	66.597
		SIF-IDF	0.000	34.706	76.542
Bms-1	10	EMO-AddItem	6.500	4.897	4.613
		Algo1.a	40.000	20.929	4.111
		WSDA	0.000	14.295	0.841
		SIF-IDF	0.000	8.257	0.532
	20	EMO-AddItem	21.000	16.113	9.920
		Algo1.a	50.000	38.219	6.538
		WSDA	0.000	33.479	1.435
		SIF-IDF	0.000	16.033	0.859
Bms-2	10	EMO-AddItem	0.000	4.873	3.675
		Algo1.a	20.000	9.769	2.518
		WSDA	0.000	6.738	0.661
		SIF-IDF	0.000	2.707	0.190
	20	EMO-AddItem	0.000	8.750	5.738
		Algo1.a	40.000	20.563	3.335
		WSDA	0.000	8.302	0.955
		SIF-IDF	0.000	5.829	0.343
Retail	10	EMO-AddItem	0.000	0.031	0.222
		Algo1.a	0.000	0.031	0.222
		WSDA	0.000	0.061	0.129
		SIF-IDF	0.000	3.735	1.000
	20	EMO-AddItem	0.000	0.043	0.524
		Algo1.a	5.000	0.061	0.382
		WSDA	5.000	0.215	0.284
		SIF-IDF	0.000	4.730	1.594

In Table 3, we may observe that EMO-AddItem can achieve better results on the performance in the form of knowledge distortion, although this was achieved at the cost of greater data distortion degrees. As indicated previously, the main goal of data sanitization is to hide sensitive rules with the least damage to the non-sensitive knowledge, and the data distortion is used for an illustration. When sharing data between different organizations, a minimum data distortion degree may be specified in the agreement to ensure a reasonable data quality. When the minimum threshold can be satisfied, we should seek the lowest damage to the non-sensitive knowledge. Actually, the result of EMO-AddItem contained multiple alternative tradeoff solutions for each test case, and this brings the freedom for a user to choose other solutions if his or her preference changes. For instance, the user may select the solution which holds the lowest hiding failure or knowledge distortion but can simultaneously meet the minimum data distortion threshold specified in the sharing arrangement.

As we have discussed in Subsection 5.1, the strategy of adding items cannot ensure all sensitive rules to be hidden. The results of EMO-AddItem and 1.a in Table 3 verify this point. Although the strategy of removing items often may completely conceal all sensitive rules in most cases, Table 3 shows that it also may lead to non-zero hiding failure in some situations. For instance, in the case of 20 rules on Retail, WSDA cannot completely hide them. This is caused by the duplicate items within sensitive rules. WSDA only can hide one rule in each iteration. This provides the possibility that hiding a subsequent rule cause the confidence of an already hidden overlapping rule to be exposed above *MCT* again, due to the reduced support of its antecedent.

The algorithm 1.a selects transactions for modification in accordance to how many items in the sensitive rule’s antecedent are also contained in a transaction. It assumes that, by choosing the transaction which contains the largest subset of items in the antecedent of a sensitive rule, the impact on the database may be minimized and fewer side effects may be generated. This heuristic information is utilized by it to sort the candidate transactions for each sensitive rule and the foremost ones are selected for modification. It seems reasonable to use this heuristic information to choose transactions. However, it is not sufficient to make a decision only based on it because in some datasets most transactions hold the same number of items which appear in the antecedent part of a sensitive rule. In such a situation, the procedure of selecting transactions in the algorithm 1.a is almost run in a completely random manner.

The density of a dataset may affect the data distortion degree. Generally, in a denser dataset the number of transactions that have to be sanitized tends to be higher than a sparser one. Thus to sanitize a denser dataset may lead to a higher data distortion degree. This can be verified from the results on Mushroom in Table 3.

**Fig 4:**
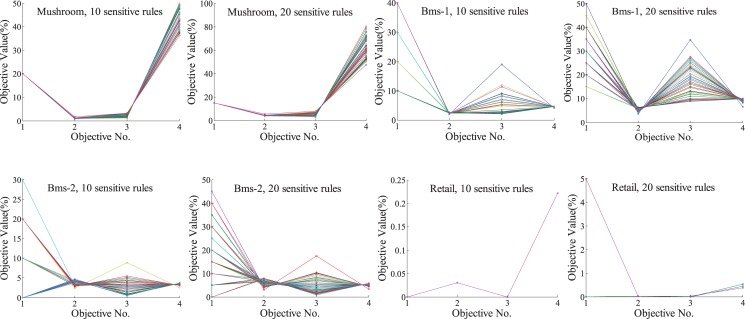
Tradeoffs exist within different side effects.

From the view of multi-objective optimization, EMO-AddItem addresses the issue as a four-objective optimization problem. In fact, tradeoffs exist within different side effects. Fig 4 shows the value path plots of obtained Pareto optimal solutions. The numbers, 1, 2, 3 and 4, denote four optimization goals. There are eight test cases in Fig 4, corresponding to the results in Table 3. As Fig 4 indicates, on Bms-1 and Bms-2, EMO-AddItem can find a good spread of solutions. The diversities on the 1^*st*^ objective (hiding failure) and 3^*rd*^ objective (ghost rules) are great. The 4^*th*^ objective, i.e. the data distortion degree, varies little on Bms-1 and Bms-2. It demonstrates that the data distortion degree will not increase so much even if we choose a preferred solution which gives the priority to lower hiding failure or knowledge distortion. However, for Mushroom, more diversity is reflected in the fourth objective, data distortion. In such a situation, we have to consider the fluctuation of data distortion when choosing different solutions.

#### Results with different *MCT* levels

Different *MCT* levels may influence the number of strong rules. A lower *MCT* level will produce more rules after the mining process. Four levels of *MCT* (20%, 30%, 40% and 50%) were tried on the Bms-1 dataset. The same 10 sensitive rules with high confidence values were used in experiments. This required that each sensitive rule held a confidence value above 50%.

**Fig 5:**
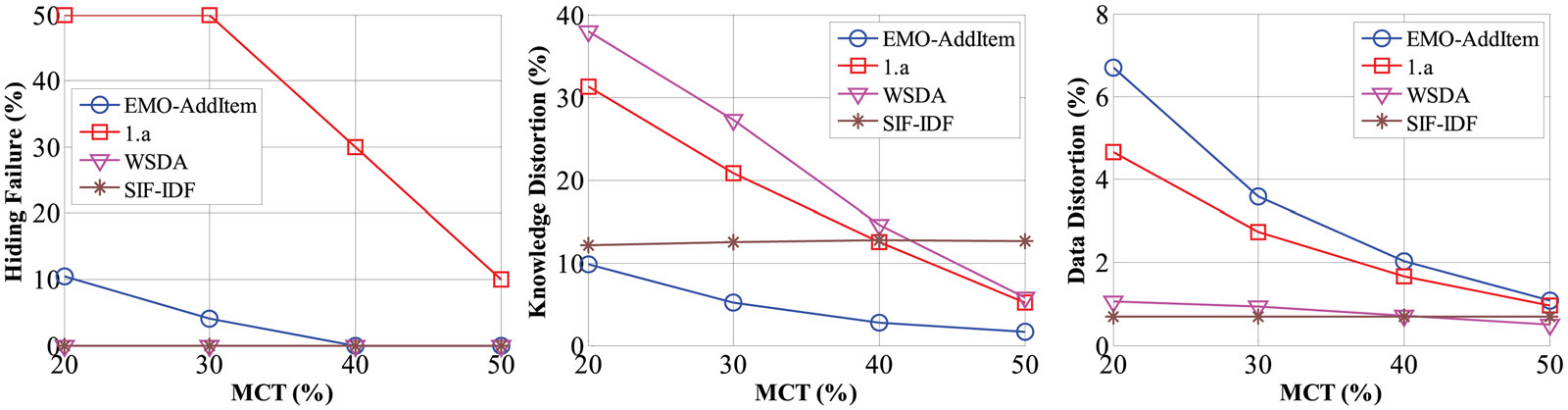
Side effects with increasing *MCT* levels.

As Fig 5 indicates, side effects on hiding failure, knowledge distortion and data distortion decrease accordingly with increasing *MCT* levels for both algorithms. When the level of *MCT* rises, the number of transactions which have to be sanitized to hide a sensitive rules will be reduced, according to Eq 2. The fewer the sanitized transactions, the smaller side effects produced. Thus, side effects of all methods become smaller when increasing *MCT* levels.

Again, the methods based on removing items, i.e., WSDA and SIF-IDF, conceal sensitive rules completely for this group of experiments. EMO-AddItem perform sanitization with the least impact on knowledge distortion, at the cost of greater degrees of data distortion. We may notice that side effects of SIF-IDF almost remain constant when increasing *MCT* levels. SIF-IDF is a support-based sanitization approach. It hides a rule by reducing its support below *MST*, and the confidence is not utilized. Therefore, to increase *MCT* levels does not affect the degree of data distortion (or the number of sanitized transactions) for SIF-IDF. However, lowered *MCT* levels may produce more rules. The almost constant degree of knowledge distortion for SIF-IDF reflects that the absolute number of missing non-sensitive rules or ghost rules has been raised along with decreasing *MCT* levels.

## 7 Conclusion

In this paper, we adopted the technique of evolutionary multi-objective optimization to solve the association rule hiding problem. A new hiding solution named as EMO-AddItem is proposed. The hiding strategy is to insert items into the transactions, which partially (or not) support the antecedent part and not fully support the consequent part of a sensitive rule at the same time, so as to decrease the confidence of the sensitive rule below a specified thresh-old, i.e., *MCT*. Taking it as a multi-objective optimization problem, the side effects, including hiding failure, missing non-sensitive rules, ghost rules and data distortion are formulated as optimization objectives. The goal is to find the optimal subset of transactions for sanitization to hide all sensitive rules and simultaneously minimize side effects accompanied. Because this problem includes four optimization goals, HypE, a fast hypervolume-based algorithm dedicated to many-objective optimization, is utilized to drive the evolution process forward. The performance of the proposed approach was empirically compared with the algorithm 1.a, WSDA and SIF-IDF on real-world datasets. The results demonstrated that EMO-AddItem can perform the sanitization task with fewer knowledge distortion for most test cases. In the future work, we will investigate how the strategy of adding items can be combined with strategy of removing items in a blocking-based way. The blocking-based hiding way sanitizes a database by replacing some items with unknown [9, 10], which is considered to bring less damage to the database itself than the distortion-based way. It hides sensitive rules by increasing the uncertainties of their supports and confidences. In addition, EMO might be extended to other privacy preserving problems on which the tradeoff often occurs.

## Supporting Information

S1 Data Experiment result data. (ZIP)
